# Supplementary material for: Gross Total Resection Promotes Subsequent Recovery and Further Enhancement of Impaired Natural Killer Cell Activity in Glioblastoma Patients
Source: Brain Sci. 2022 Aug 27;12(9):1144. doi: 10.3390/brainsci12091144 (PMC9496976; doi:10.3390/brainsci12091144)
Supplement: Supplementary file 1 [file brainsci-12-01144-s001.zip › Revised_Supplementary Figures S1-S3.pdf]

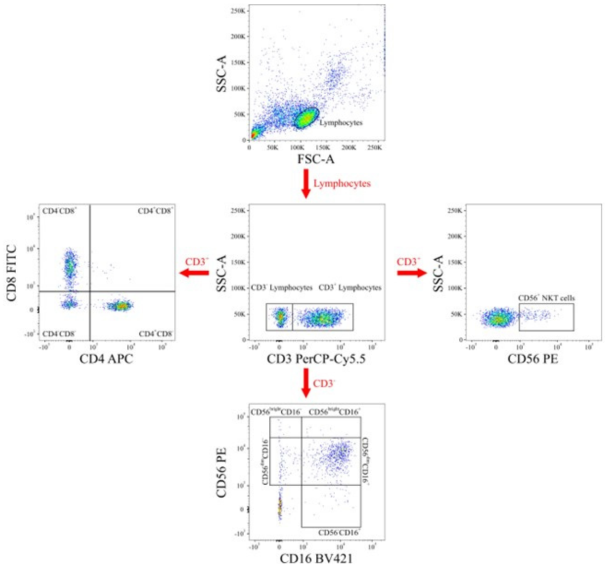

Figure S1. Gating strategy for identification of NK cell and T cell subsets.

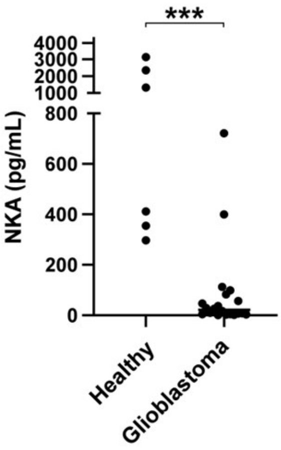

Figure S2. NKA was significantly downregulated in glioblastoma patients

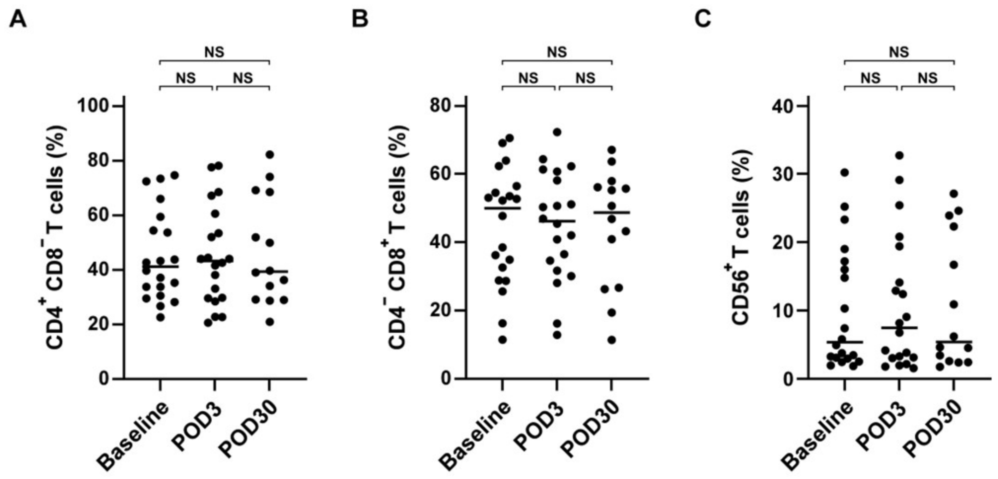

Figure S3. T cell subsets were not redistributed before after cranial surgery.
